# Central ventilatory and cardiovascular actions of trout gastrin-releasing peptide (GRP) in the unanesthetized trout

**DOI:** 10.1242/bio.20135553

**Published:** 2013-07-30

**Authors:** Jean-Claude Le Mével, Frédéric Lancien, Nagi Mimassi, Marc Kermorgant, J. Michael Conlon

**Affiliations:** 1 Université Européenne de Bretagne, Université de Brest, INSERM UMR1101, Laboratoire de Traitement de l'Information Médicale, Laboratoire de Neurophysiologie, SFR ScInBioS, Faculté de Médecine et des Sciences de la Santé, 22 Avenue Camille Desmoulins, CS 93837, 29238 Brest Cedex 3, CHU de Brest, France; 2 Department of Biochemistry, College of Medicine and Health Sciences, United Arab Emirates University, 17666 Al Ain, United Arab Emirates

**Keywords:** Central GRP, Intracerebroventricular injection, Hyperventilation, Hypertension, Teleost

## Abstract

Gastrin-releasing peptide (GRP), a neuropeptide initially isolated from porcine stomach, shares sequence similarity with bombesin. GRP and its receptors are present in the brains and peripheral tissues of several species of teleost fish, but little is known about the ventilatory and cardiovascular effects of this peptide in these vertebrates. The goal of this study was to compare the central and peripheral actions of picomolar doses of trout GRP on ventilatory and cardiovascular variables in the unanesthetized rainbow trout. Compared to vehicle, intracerebroventricular (ICV) injection of GRP (1–50 pmol) significantly elevated the ventilation rate (ƒV) and the ventilation amplitude (*V*AMP), and consequently the total ventilation (*V*TOT). The maximum hyperventilatory effect of GRP (*V*TOT: +225%), observed at a dose of 50 pmol, was mostly due to its stimulatory action on *V*AMP (+170%) rather than ƒV (+20%). In addition, ICV GRP (50 pmol) produced a significant increase in mean dorsal aortic blood pressure (*P*_DA_) (+35%) and in heart rate (ƒH) (+25%). Intra-arterial injections of GRP (5–100 pmol) were without sustained effect on the ventilatory variables but produced sporadic and transient increases in ventilatory movement at doses of 50 and 100 pmol. At these doses, GRP elevated *P*_DA_ by +20% but only the 50 pmol dose significantly increased HR (+15%). In conclusion, our study suggests that endogenous GRP within the brain of the trout may act as a potent neurotransmitter and/or neuromodulator in the regulation of cardio-ventilatory functions. In the periphery, endogenous GRP may act as locally-acting and/or circulating neurohormone with an involvement in vasoregulatory mechanisms.

## Introduction

Some regulatory peptides that were first discovered within the gastrointestinal system are also produced in brain nuclei and can act within the brain as neurotransmitters and/or neuromodulators to control various physiological functions. In mammals, including humans, gastrin-releasing peptide (GRP), a neuropeptide originally isolated from porcine stomach (McDonald et al., 1979), comprises 27 amino acid residues and shares a seven amino acid COOH-terminal sequence with bombesin, a tetradecapeptide isolated from the skin of the frog *Bombina bombina* (Erspamer et al., 1970; Anastasi et al., 1971; Spindel et al., 1984). However, it has been shown that bombesin does not represent the amphibian GRP and the peptide is not present in either mammalian or fish tissues (Conlon et al., 1991). Consequently, most previous studies in the past relating to the physiological role of GRP in vertebrates are of limited value since they were carried out using bombesin. It is always preferable to study the effects of a naturally occurring peptide in its species of origin. GRP binds preferentially to a G-protein coupled receptor designated as GRP receptor (GRPR, also called BB2) (Lehmann and Beglinger, 2006; Roesler and Schwartsmann, 2012). GRP-like immunoreactivity is widely distributed throughout the mammalian gastrointestinal tract and GRP affects several gastrointestinal functions including motility, and endocrine and exocrine secretions. In addition, GRP is also present in a range of tumor cell lines (Lehmann and Beglinger, 2006). High levels of GRP mRNA, GRP immunoreactivity and GRPR mRNA are also found in brain including the cortical areas, the hippocampus, the amygdaloid nuclei, the hypothalamic paraventricular nucleus and in the brainstem area, notably the nucleus tractus solitary (NTS) and the nucleus ambiguus (Moody and Merali, 2004; Roesler and Schwartsmann, 2012). GRP and its receptor appear to be critical in the regulation a variety of brain functions including, memory, stress, neuroendocrine functions, and behaviour (Moody and Merali, 2004; Roesler and Schwartsmann, 2012). However, the physiological role of GRP in cardioventilatory regulation is incompletely understood since most functional studies have been carried out using bombesin rather than GRP. In rats, bombesin injection within the brain elicits hypothermia (Brown et al., 1977a), hyperglycemia (Brown et al., 1977b), elevates blood pressure and causes bradycardia (Fisher et al., 1985). In the anesthetized cat, bombesin acts in the brain to stimulate ventilation (Holtman et al., 1983). Recent data demonstrate, however, that microinjection of GRP into the NTS in urethane-anesthetized rats increases ventilation through stimulation of tidal volume but this injection was without effect on blood pressure or heart rate (ƒH) (Aliev et al., 2012).

The amino acid sequence of GRP in trout is shorter than those of mammalian GRPs by four residues indicating that evolutionary pressure to conserve the full sequence of the peptide has not been strong (Jensen and Conlon, 1992). Nevertheless, the amino acid domain at the COOH-terminus of the trout peptide is the same as the corresponding region in mammalian GRPs but shows three substitutions compared with bombesin (Jensen and Conlon, 1992). GRP transcripts and GRP immunoreactive peptides are also widely distributed in the gastrointestinal tract and central nervous system (CNS) of various fish, including elasmobranchs (Conlon et al., 1987; Bjenning et al., 1991) and teleosts (Jensen and Conlon, 1992; Volkoff et al., 2000; Xu and Volkoff, 2009). In the rainbow trout brain, the highest density of GRP-like immunoreactivity is observed in the hypothalamic area were positive cell bodies were detected. Immunoreactive fibers are widely distributed in the diencephalon at the level of nucleus preopticus (NPO), midbrain and hindbrain including the medulla oblongata (Cuadrado et al., 1994). In the goldfish, a high density of GRP binding sites was also found in the preoptic region of the hypothalamus (Himick et al., 1995). However, only few *in vivo* studies have evaluated the peripheral or central actions of GRP in teleosts. Infusions of bombesin and GRP stimulate acid gastric secretion and motility in cod (Holstein and Humphrey, 1980; Holmgren and Jönsson, 1988) and bombesin inhibits intestinal secretion in this species (Jensen and Holmgren, 1985). In addition, bombesin has been shown to potentiate the *in vitro* contractile effect of acetylcholine on trout and cod gastric smooth muscle (Thorndyke and Holmgren, 1990). GRP peptides appear to be involved in satiety since bombesin decreases feeding following intraperitoneal (IP) injections in carp (Beach et al., 1988) and after both IP and intracerebroventricular (ICV) injections in goldfish (Himick et al., 1995). The cardiovascular effects of GRP in fish are poorly understood. *In vivo*, in the spiny dogfish *Squalus acanthias*, bombesin increases the dorsal aortic blood pressure and provokes a bradycardia that might be reflexly mediated (Nilsson and Holmgren, 1992).

The main goal of this study is to provide insight into the CNS effects of GRP on ventilatory and cardiovascular functions in the unanesthetized rainbow trout, *Oncorhynchus mykiss*. To this end, we have analyzed the effects of ICV administration of synthetic replicate of trout GRP on ventilation rate (ƒV), ventilation amplitude (*V*AMP), dorsal aortic blood pressure (*P*_DA_) and ƒH. Additionally, the central actions of the peptide were compared with its effects after intra-arterial (IA) injection.

## Materials and Methods

### Peptides and chemicals

Trout GRP (SENTGAIGKVFPRGNHWAVGHLM.NH_2_) (Jensen and Conlon, 1992) was synthesized by GL Biochem (Shanghai, China) and purified to near homogeneity (>98% purity) by reversed-phase HPLC. The identity of the peptide was confirmed by electrospray mass spectrometry. The peptide was diluted in vehicle and stored in stock solution at −25°C. For injections, GRP was diluted to the desired concentration with Ringer solution (vehicle) immediately prior to use. The composition of the Ringer solution was (in mM): NaCl 124, KCl 3, CaCl_2_ 0.75, MgSO_4_ 1.30, KH_2_PO_4_ 1.24, NaHCO_3_ 12, glucose 10 (pH: 7.8). All solutions were sterilized by filtration through 0.22 µm filters (Millipore, Molsheim, France) before injection.

### Animals

Adult rainbow trout *Oncorhynchus mykiss* (mean ± s.e.m., body mass = 260±4.2 g, *n* = 32) of both sexes were purchased locally and transferred in a well-oxygenated and thermostatically controlled water tank to the laboratory. All the fish were kept in a 1,000 liter tank containing circulating dechlorinated, aerated tap water (11–12°C), under a standard photoperiod (lights on 09:00–20:00 h). The fish were allowed at least three weeks to acclimate under these conditions before the experiments were started. Experimental protocols were approved by the Regional Ethics Committee in Animal Experiments of Brittany, France (permit number 07).

### Experimental procedures

All surgical procedures were made under tricaine methane sulfonate (3-amino-benzoic acid ethyl ester; 60 mg/l in tap water buffered with NaHCO_3_ to pH = 7.3–7.5) anesthesia. The techniques used for placement of the electrocardiographic (ECG) electrodes, placement of the buccal catheter, cannulation of the dorsal aorta and insertion of the ICV microguide have previously been described in detail (Le Mevel et al., 1993; Lancien et al., 2004). Briefly, two ECG AgCl electrodes (Comepa, 93541 Bagnolet, France) were subcutaneously implanted ventrally and longitudinally at the level of the pectoral fins. The incision was sutured across the electrodes and the leads were sutured to the skin. The dorsal aorta was cannulated with a PE-50 catheter (Clay Adams, Le Pont De Claix, France) (Soivio et al., 1972). A flared cannula (PE-160) was inserted into a hole drilled between the nares such that its flared end was resting against the roof of the mouth. This cannula was used to record any changes in buccal ventilatory pressure (Holeton and Randall, 1967). The absence of a neocortex in fish allows the accurate placement of the ICV microguide under stereomicroscopic guidance. A 25-gauge needle fitted with a PE-10 polyethylene catheter was inserted between the two habenular ganglia and descended into the third ventricle until its tip lay between the two preoptic nuclei (Le Mével et al., 2009). An obturator was placed at the end of the PE-10 tubing and the cranial surface was covered with hemostatic tissue followed by light quick-curing resin. After surgery, the animals were force-ventilated with dechlorinated tap water and, following recovery of opercular movements, were transferred to a 6 liter blackened chamber supplied with dechlorinated and aerated tap water (10–11°C) that was both recirculating and through-flowing. Oxygen partial pressure within the water tank (*P*wO_2_) and pH were continuously recorded and maintained at constant levels (*P*wO_2_ = 20 kPa; pH = 7.4–7.6). A small horizontal aperture was made along the upper edge of the chamber in order to connect the ECG leads to an amplifier and to connect the dorsal aorta and the buccal cannula to pressure transducers. This aperture also permitted ICV and IA injections of peptides without disturbing the trout.

The trout were allowed to recover from surgery and to become accustomed to their new environment for 48–72 h. Each day, the general condition of the animals was assessed by observing their behaviour, checking the ventilatory and the cardiovascular variables, and measuring their hematocrit. Animals that did not appear healthy, according to the range of values detailed in our previous studies, were discarded. After stable ƒV, *V*AMP, *P*_DA_ and ƒH were maintained for at least 90 min, parameters were recorded for 30 min without any manipulation in control experiments.

### Intracerebroventricular administration of GRP

The injector was introduced within the ICV guide prior to the beginning of a recording session which lasted 30 min. All injections were made at the 5^th^ min of the test but the injector was left in place for a further 5 min to allow for complete diffusion of the agent and to minimize the spread of substances upwards in the cannula tract. The fish received first an ICV injection of vehicle (0.5 µl) and 30 min later, an ICV injection of GRP (1, 5 and 50 pmol in 0.5 µl). Previous control experiments using two ICV injections 30 min apart have shown no time-dependent changes in the measured variables using this protocol (Le Mével et al., 2009). The animals received no more than two ICV injections of peptide per day with a delay of at least five hours between the injections. No single fish was studied for more than 2 days and control experiments revealed that there was no significant change in performance over this period.

### Intra-arterial administration of GRP

Five min after the beginning of the recording session, 50 µl of vehicle, or trout GRP at doses of 5, 50 and 100 pmol was injected through the dorsal aorta and immediately flushed by 150 µl of vehicle.

### Data acquisition and analysis of the ventilatory and the cardiovascular variables

The ECG electrodes were connected to a differential amplifier (band pass: 5–50 Hz; Bioelectric amplifier, Gould and Nicolet, 91942 Courtaboeuf, France) and a stainless steel bar was immersed in the water of the tank to act as a reference electrode. The aortic cannula and the buccal catheter were connected to P23XL pressure transducers (band-pass: 0–15 Hz; Gould and Nicolet). These pressure transducers were calibrated each day using a static water column. At the beginning of the experiments, the zero-buccal pressure level was set electronically. The zero-blood pressure level was set to the level of water surface. The output signals from the devices were digitalized at 1000 Hz and visualized on the screen of a PC using PowerLab 4/30 data acquisition system (ADInstruments, Oxford, England) and LabChart Pro software (v.7.0; ADInstruments, Oxford, England) during the 30-min recording period and the data were stored on a disc. The time-series related to the ventilatory, the pulsatile *P*_DA_ and the ECG signals were then processed off-line with custom-made programs written in LabView 6.1 (Laboratory Virtual Instrument Engineering Workbench, National Instruments, Austin, USA). The ventilatory and cardiovascular variables were calculated as previously described (Lancien et al., 2004; Le Mével et al., 2007). Segments free of any movement artifacts on the ventilatory signal were selected and ƒV (breaths min^−1^) and *V*AMP (arbitrary units, a.u.) were determined. The ƒV was calculated from the first harmonic of the power spectrum of the ventilatory signal using the fast Fourier transformation. *V*AMP was calculated from the difference between the maximal abduction phase and the maximal adduction phase for each ventilatory cycle. Spontaneous coughings were excluded from this analysis. The net effect of the changes in ƒV and *V*AMP on ventilation was estimated according to the formula *V*TOT = ƒV*VAMP where *V*_TOT_ (a.u.) is total ventilation. The mean *P*_DA_ (kPa) was calculated from the pulsatile *P*_DA_ as the arithmetic mean between the systolic blood pressure and the diastolic blood pressure and the ƒH (beats min^−1^) was determined from the ECG signal. All calculations for mean ƒV, *V*AMP, V_TOT_, *P*_DA_ and ƒH were made for the pre-injection period (0–5 min) and for five post-injection periods of 5 min for each trout and the results were averaged for trout subjected to the same protocol.

### Statistical analysis

Data are expressed as means ± s.e.m. for each 5-min period on time course histograms. In the figures, and text, data refer to absolute values, or as maximal percentage changes from baseline (pre-injection) values. The absolute data values were analyzed initially using two-way ANOVA (treatments and time) followed by the Bonferroni post hoc test for comparisons between groups. Within each group of trout receiving peptide, when the overall preceding two-way ANOVA analysis demonstrated statistically significant differences compared to vehicle-injected trout, Dunnett's test was used for comparisons of post-injection values with pre-injection values. The criterion for statistical difference between groups was *P*<0.05. The statistical tests were performed using GraphPad Prism 5.0 (GraphPad, San Diego, CA).

## Results

### Ventilatory and cardiovascular responses to central GRP

**Fig. 1.**
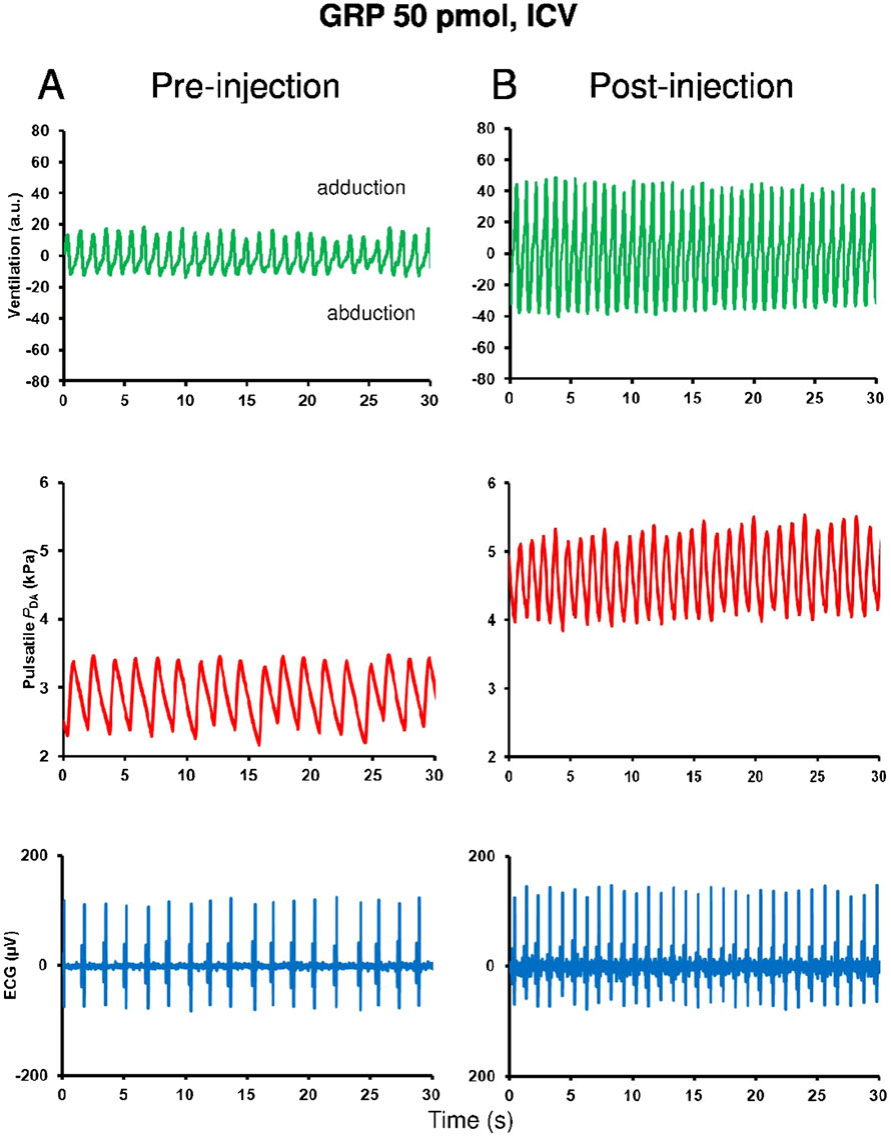
Recording traces of 30 sec duration in the same unanesthetized trout illustrating the changes observed in ventilatory movements (ventilation), pulsatile dorsal aortic blood pressure (*P*_DA_) and electrocardiographic (ECG) signals between the pre-injection period (0–5 min) (**A**) and the post-injection period (20–25 min) (**B**) after intracerebroventricular (ICV) injection of 50 pmol GRP. Note that, compared with the pre-injection period, the ICV injection of GRP produces an increase in the ventilation rate and amplitude, and an elevation of blood pressure and heart rate.

Fig. 1 illustrates recordings for 30 sec in a single trout of the ventilatory, blood pressure and ECG signals taken during the pre-injection period (Fig. 1A) and during the 20–25 min post-injection period (Fig. 1B) after ICV injection of 50 pmol GRP. Comparing the post-injection and the pre-injection signals, GRP caused a marked elevation in ƒV and *V*AMP. Concurrently, ICV injection of GRP produced an increase in systolic and diastolic *P*_DA_ and consequently GRP caused an increase in mean *P*_DA_. GRP also elicited a tachycardia.

**Fig. 2.**
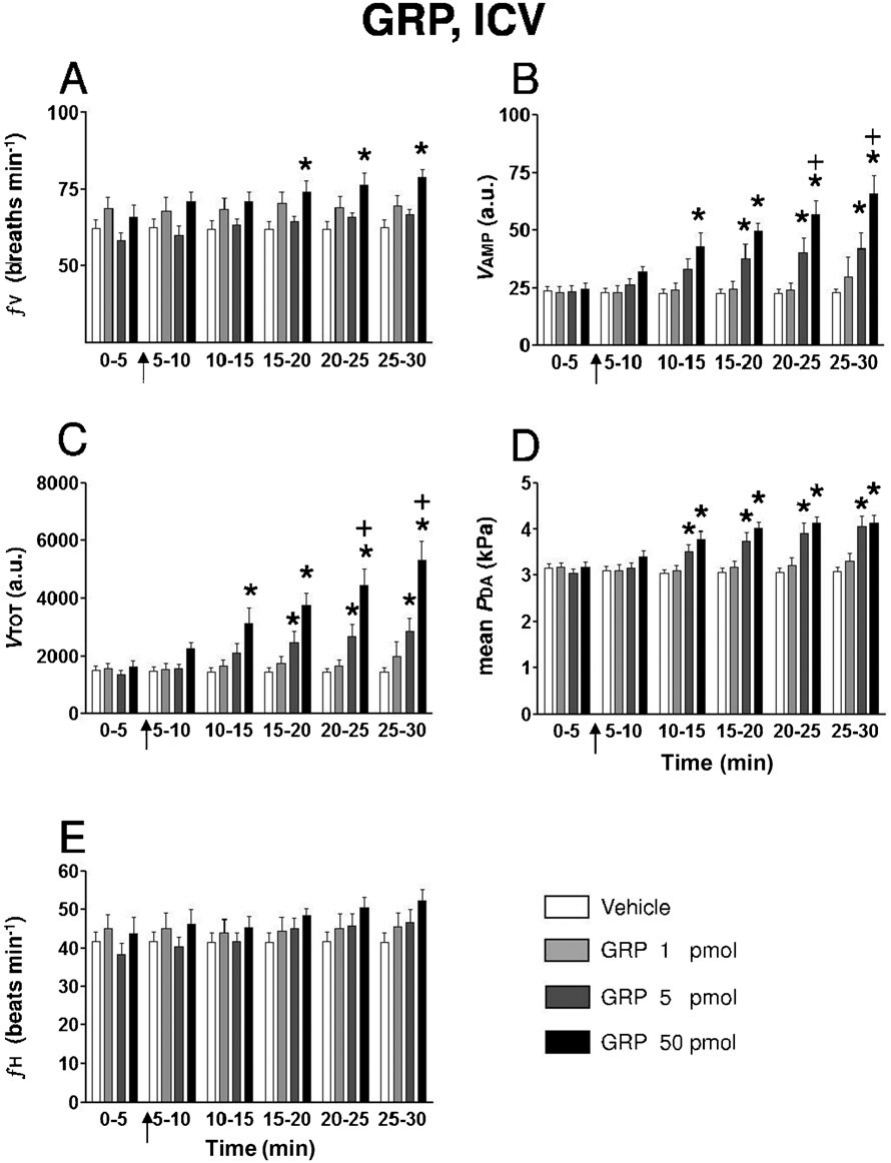
Histograms showing the time-course of the effects of intracerebroventricular (ICV) injection of (1) 0.5 µl of vehicle (*n* = 21), (2) 1 pmol GRP (*n* = 7), (3) 5 pmol GRP (*n* = 12) and (4) 50 pmol GRP (*n* = 9) on (**A**) ƒV , (**B**) *V*AMP, (**C**) *V*TOT, (**D**) mean *P*_DA_ and (**E**) ƒH in unanesthetized trout. The arrows indicate when the injection was given. n, number of trout. *P<0.05 *vs* vehicle at corresponding post-injection period and *vs* pre-injection value. +*P*<0.05 *vs* GRP 5 pmol.

Fig. 2 summarizes the time course of effects observed in the ventilatory and cardiovascular variables following ICV injections of vehicle or a range of doses (1–50 pmol) of GRP. ICV injection of vehicle produced no significant change in the ventilatory and cardiovascular variables compared to pre-injection values. Compared with ICV injection of vehicle, only the highest dose of GRP (50 pmol) evoked a significant increase in ƒV after a latency of about 10–15 min (Fig. 1A). GRP produced a dose and time-dependent increase in *V*AMP. The threshold dose for a significant stimulatory effect of GRP on *V*AMP was 5 pmol (Fig. 1B). The effect on *V*AMP of GRP (50 pmol) was significantly greater than that of GRP (5 pmol) during the 20–30 minutes period of the recording. Consequently, the net effect of the peptide was a hyperventilatory response involving a gradual, significant and dose-dependent increase in *V*TOT (Fig. 2C). The most pronounced action of GRP was evoking hyperventilation through an increase in *V*AMP instead of ƒV. For instance at a dose of 50 pmol, during the 25–30 min post-injection period when *V*_TOT_ was maximal and increased by about 225% from baseline value (5304±681 *vs* 1622±207, *P*<0.05; Fig. 2C), the elevation of ƒV expressed as a percentage from pre-injection value was only 20% (78.9±2.2 *vs* 65.7±4.70 breaths min^−1^, *P*<0.05) while the change in *V*AMP was 170% (66.2±7.9 *vs* 24.5±3.9 a.u., *P*<0.05) (Fig. 2A,B). The actions of GRP on these ventilatory variables persisted until the end of the recording. After ICV injection, neither vehicle nor 1 pmol GRP evoked any cardiovascular action. However, GRP at doses of 5 and 50 pmol produced quite similar significant and sustained increases in *P*_DA_ (maximum increase; 50 pmol: +30%; Fig. 2D) but the increase in ƒH did not reach the level of statistical significance in the two-way ANOVA test (maximum increase 50 pmol: +20%; Fig. 2E).

### Ventilatory and cardiovascular responses to peripheral GRP

**Fig. 3.**
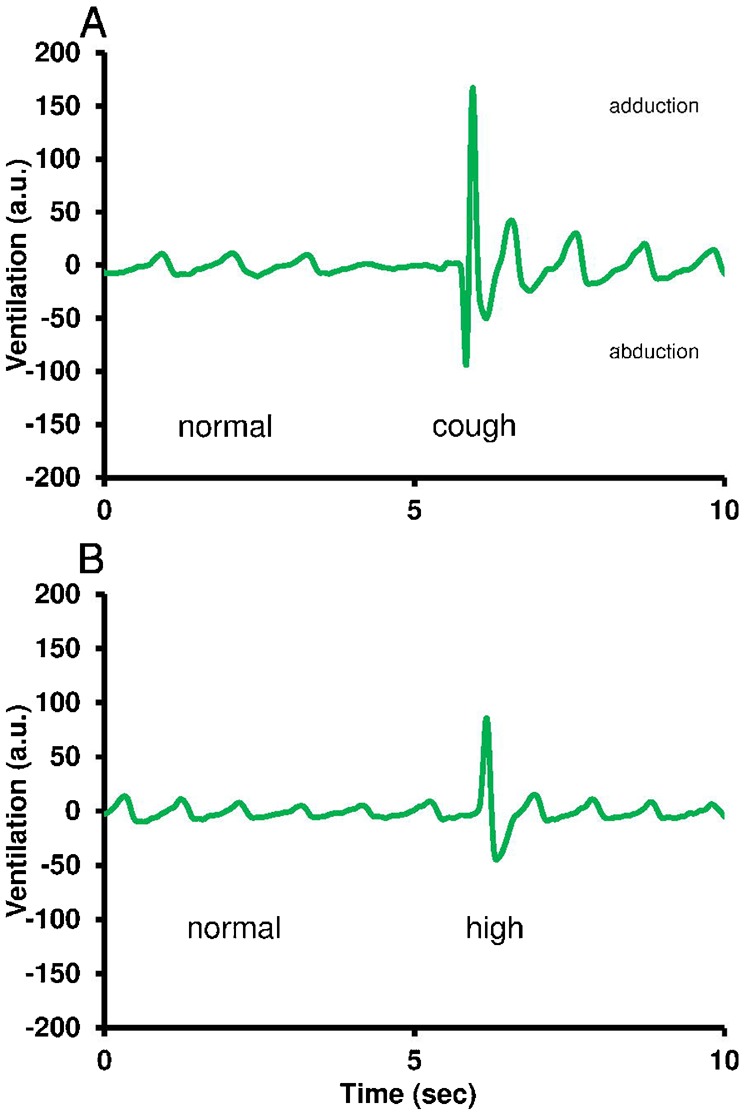
Experimental traces showing the various patterns of ventilatory movements in the unanesthetized trout (**A**) during the pre-injection period and (**B**) after intra-arterial injection of GRP (50 pmol). ‘normal’, normal ventilatory pattern; ‘cough’, spontaneous occurring cough; ‘high’, sporadic high increase in the adduction and abduction phases of ventilatory movement. This last pattern was only seen after an intra-arterial injection of GRP.

**Fig. 4.**
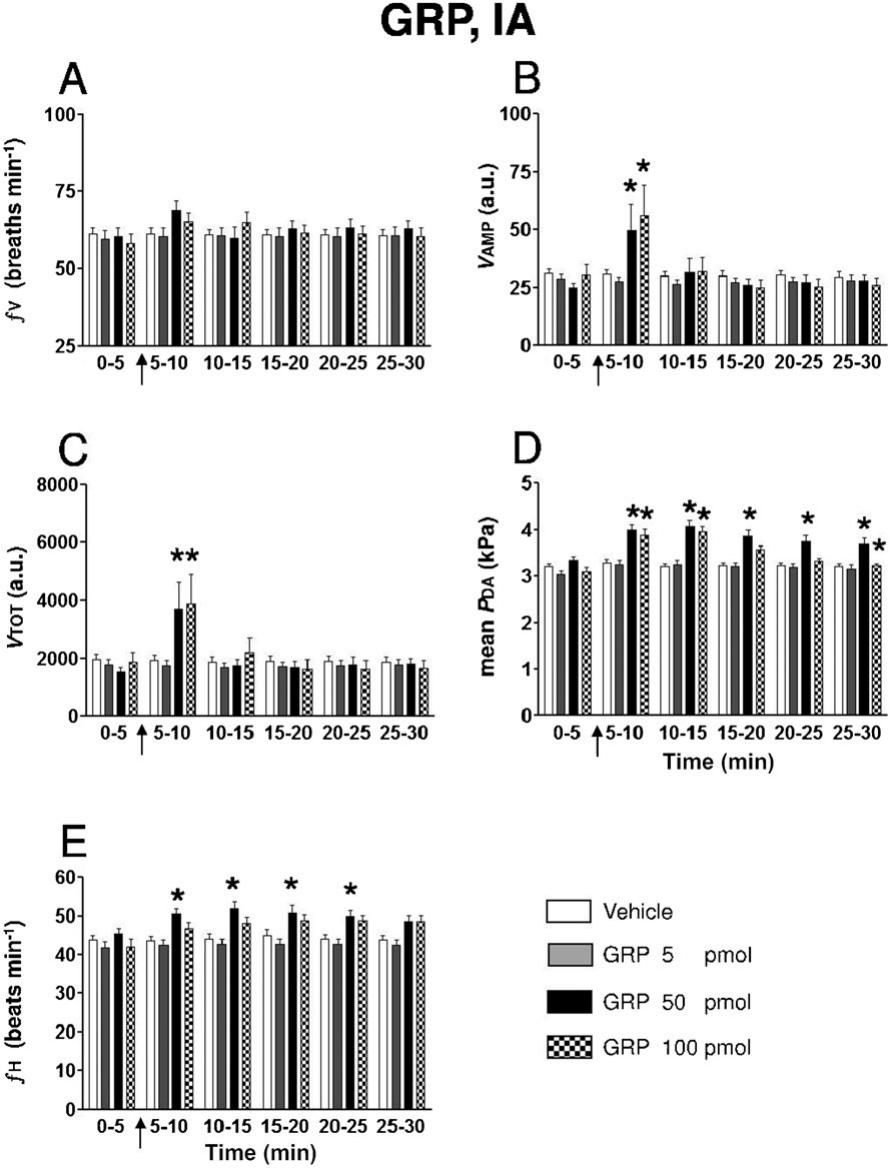
Histograms showing the time-course of the effects of intra-arterial injection of (1) 50 µl of vehicle (*n* = 25), (2) 5 pmol GRP (*n* = 19), (3) 50 pmol GRP (*n* = 12) and (4) 100 pmol GRP (*n* = 11) on (**A**) ƒV , (**B**) *V*AMP, (**C**) *V*TOT, (**D**) mean *P*_DA_ and (**E**) ƒH in unanesthetized trout. The arrow indicates when the injection was given. n, number of trout. *P<0.05 *vs* vehicle at corresponding post-injection period and *vs* pre-injection value.

After IA injection at doses of 50 and 100 pmol, GRP enhanced the adduction/abduction phase of some ventilatory movements. These sporadic events occurred 60–90 sec after the injection and lasted for at least 5 min. This high ventilatory response was clearly recognized from the normal regular ventilatory movements and from spontaneous occurring cough (Fig. 3). On average, *V*AMP and *V*_TOT_, but not ƒV, significantly increased by two-fold during the 5–10 min post-injection period (Fig. 4A,B,C). In addition, peripherally injected GRP at doses of 50 and 100 pmol elicited an overall significant hypertensive response, an effect which was not dose-dependent in this dose range (maximum increase in *P*_DA_ ; 50 pmol: +20%). The increase in ƒH was only significant for the 50 pmol dose (Fig. 4D; maximum increase in ƒH; 50 pmol: +15%).

## Discussion

To the best of our knowledge, this is the first study to examine the central effect of picomolar doses of intracerebroventricularly administered GRP on the cardioventilatory system of any vertebrate. Native GRP in trout produces a hyperventilation mostly through a stimulatory action of *V*AMP. In addition, GRP elevates *P*_DA_ without significantly enhancing ƒH. When injected intra-arterially, doses of GRP that elicited sustained stimulatory ventilatory effects when administered intracerebroventricularly or even larger doses evoke only sporadic and transient increase in the amplitude of some ventilatory movements. This observation strongly supports the assumption that ICV injection of GRP causes a sustained hyperventilation through an action within the central nervous system. GRP elicits slight hypertensive and tachycardic responses after systemic injection. Taken together these cardiovascular results favour the concept that exogenous GRP can act through neurogenic mechanisms but also through some direct or indirect peripheral pathways to affect blood pressure and ƒH.

### Ventilatory and cardiovascular actions of intracerebroventricularly administered GRP

Our results demonstrating that third ventricle injection of trout GRP in trout causes hyperventilation and hypertension without significant change in ƒH may be compared to previous cardioventilatory studies conducted in mammals using bombesin instead of a mammalian GRP. In the anesthetized cat, bombesin (0.62–6.2 nmol) administered into the cisterna magna increases ventilation through a primary action on tidal volume (Holtman et al., 1983). In the anesthetized rat, ICV bombesin (0.1–10 µg, approximately 0.6–6 nmol) induces a rapid increase in tidal volume with variable action on ƒV. The net effect of bombesin is an increase in minute ventilation (Niewoehner et al., 1983; Hedner et al., 1985). In these animals, ICV bombesin does not change blood pressure but decreases ƒH (Hedner et al., 1985). In addition, local injection of the peptide into the nucleus ambiguus, but not in several other nuclei of the brainstem, reproduces the stimulatory effect of ICV bombesin on ventilation (Hedner et al., 1985). These stimulatory effects of bombesin in rats seem to depend on intact afferent vagal innervations (Hedner et al., 1985). Additionally, ICV bombesin induced a qualitative change in breathing pattern. This change consisted of periodic inspiratory sighs in which the tidal volume was two to four times greater than normal (Niewoehner et al., 1983). Sighing response was specific for the peptide since ICV injection of saline, or a variety of neuropeptides including substance P (SP), were without action (Niewoehner et al., 1983). In unanesthetized rats, Fisher et al. demonstrate that ICV bombesin produces dose-dependent elevations of blood pressure and reduction of ƒH (Fisher et al., 1985). The increase in blood pressure was probably mediated through alpha-adrenergic receptors while the bradycardia was probably due to cardiac parasympathetic nervous activation.

The receptor site(s) initiating cellular transduction mechanisms after GRP injection cannot be deduced from the experiments in which the peptide was injected into the third cerebral ventricle. Nevertheless, neuroanatomical prerequisites and some neurophysiological data exist that might explain the ventilatory and cardiovascular responses following the ICV injection of GRP in trout. GRP was injected within the third ventricle in close proximity to the hypothalamic region, a diencephalic region where the highest density of GRP-like immunoreactive cell bodies and fibers can be observed (Cuadrado et al., 1994) and where high density of GRP binding sites were also localized (Himick et al., 1995). Therefore, it is reasonable to assume that the exogenous peptide may mimic some action of the endogenous GRP. Some GRP-like immunoreactive cell bodies located within the diencephalon show processes that enter into contact with the cerebrospinal fluid (CSF) (Cuadrado et al., 1994). This neuroanatomical singularity suggests that these neurons can sense the composition of the cerebrospinal fluid and/or release their products within the ventricular system. Exogenous GRP within the third ventricle may stimulate GRP neurons or other neurons that project their axons to the cardioventilatory centers within the brainstem (Le Mével et al., 2012). In the goldfish, functional-anatomical studies have demonstrated the existence of a neural pathway from the preoptic area to the dorsal motor nucleus of the vagus controlling concomitantly ventilation and ƒH (Hornby and Demski, 1988). The neurochemicals involved in this pathway were not determined. Arginine vasotocin and isotocin neurons from the NPO are known to project to the medulla oblongata (Batten et al., 1990; Saito et al., 2004) where these two nonapeptides might be good candidates for possible neurogenic cardioventilatory effects. Consistent with this view, centrally administered bombesin activates spinally projecting paraventricular nucleus (PVN) neurons in the anesthetized rat (Tanaka et al., 2012). The PVN in mammals is a nucleus homologous to the teleostean NPO. Another alternative route of action is that exogenous GRP may diffuse within the CSF to reach receptor sites within the brainstem. The time course of the responses to ICV GRP is consistent with this possibility. *V*AMP starts to rise 10–15 min after the ICV administration. This time may reflect the amount of time that it takes for ICV GRP to fully occupy the available GRPR. Clearly, more refined experimental work is needed to define the pathways involved in GRP-induced cardioventilatory effects after ICV injection.

### Ventilatory and cardiovascular actions of peripherally administered GRP

In the anesthetized rat, intravenous injection of bombesin evokes sighs, reduces the breathing rate but augments tidal volume and increases the blood pressure and ƒH (Kaczyńska and Szereda-Przestaszewska, 2009; Kaczyńska and Szereda-Przestaszewska, 2011). The sigh response was consistent with some results previously described after central injection of the peptide (Niewoehner et al., 1983). The effects of GRP or bombesin on ventilatory pattern after peripheral injection have never been described in fish. The episodic large and clearly distinctive adduction/abduction ventilatory movements occurring after peripheral injection of GRP might be due to activation of peripheral receptors and neural feedback mechanisms to specific medullary centers. These sporadic and short lasting ventilatory effects of peripheral administered GRP might also be due to direct action of the peptide on critical target sites in the brain that lack the blood brain barrier. Interestingly, we never observed this pattern of ventilatory episodes for any of the previously neuropeptides tested (reviewed by Le Mével et al., 2012). The mechanisms that mediate this effect and its physiological significance, if any, are at present unclear and warrant further studies.

The cardiovascular action of GRP in fish is poorly understood. *In vivo*, in the spiny dogfish *Squalus acanthias*, IA injection of bombesin increases *P*_DA_ and provokes a bradycardia that might be reflexly mediated (Nilsson and Holmgren, 1992). In the Australian lungfish *Neoceratodus forsteri*, bombesin (10 nml/kg) decreases ƒH and reduces blood flow to the lung (Holmgren et al., 1994). In our study in trout, picomolar doses of GRP evoked a hypertensive response and a tachycardia suggesting that the cardiac baroreflex response was impaired.

### Possible physiological significance

It should be noticed that our cardio-ventilatory study was conducted using the authentic GRP whereas most of the previous works in the past are of limited physiological value as they were carried out using bombesin, a peptide that is only found in the skins of a few species of frogs (Conlon et al., 1991). The present results add GRP to the list of peptides (Le Mével et al., 2012) that act centrally as metabotropic neurotransmitters to modulate the neuronal outputs that ultimately affect the ventilatory and cardiovascular effectors in the trout model. The hyperventilatory and hypertensive actions of central GRP may be viewed as cardio-ventilatory mechanisms to protect against adverse hypoventilation and hypotension. In this sense, central GRP may be part of the neurochemical array that is involved in the hypoxic ventilatory response in fish (Perry, 2011; Porteus et al., 2011). Because the hypothalamic region in the rainbow trout brain contains the largest number of SP-immunoreactive and GRP neurons and fibers (Vecino et al., 1989; Cuadrado et al., 1994), possible interaction among both neuropeptides may be of physiological importance to permit the fine control of cardiovascular and ventilatory functions. The consequence of this possible interaction is unknown, but we have previously shown that ICV injection of SP in trout decreases *V*TOT (Le Mével et al., 2007). Further studies are clearly required to elucidate the precise location of GRP-producing elements, the distribution of GRPR, and to reveal which pathways are recruited and under which circumstances the GRP and other neuropeptidergic systems are triggered to participate in integrative and homeostatic cardio-ventilatory regulations.

In conclusion, we demonstrate for the first time in any unanesthetized animal species that exogenously administered picomolar doses of GRP in the brain exert a potent stimulatory effect on ventilation. In addition, trout GRP increases blood pressure in trout with minor change in ƒH. These results suggest that the endogenous GRP within the brain of the trout may be implicated as a neurotransmitter and/or a neuromodulator in the regulation of cardio-ventilatory functions. The systemic administration of GRP evokes a hypertensive response, tachycardia and larger episodic ventilatory movements. The mechanisms mediating these effects remain to be explored in detail.
